# Pulmonary embolism diagnosis with D-dimer levels and computed tomography

**DOI:** 10.4102/hsag.v29i0.2620

**Published:** 2024-12-05

**Authors:** Rochelle A. Kruger, Jeanetta du Plessis, Henra Muller

**Affiliations:** 1 Department of Clinical Sciences, Faculty of Health and Environmental Sciences, Central University of Technology, Bloemfontein, South Africa

**Keywords:** pulmonary embolism, computed tomography, CT pulmonary angiography, D-dimer level, contrast medium, radiation dose, COVID-19

## Abstract

**Background:**

Pulmonary embolism (PE), a common heart and blood vessel disease, causes complications such as haemodynamic instability and cardiovascular mortality. Timely diagnosis and treatment are imperative for managing this potentially life-threatening condition.

**Aim:**

The aim of this study was to establish the relationship between an elevated D-dimer level and a positive computed tomography pulmonary angiogram (CTPA), which could confirm PE in patients with chest pain and suspected PE.

**Setting:**

Data were collected at a private diagnostic radiology practice located in Bloemfontein, South Africa.

**Methods:**

Data were retrospectively collected from the Picture Archiving and Communications System (PACS).

**Results:**

Of the sampled patients (*n* = 1219), only 16.7% were diagnosed with PE after CTPA. Approximately 14% of the D-dimer-positive patient group were diagnosed with PE and, in the D-dimer-negative patient group, approximately 20% of the patients were diagnosed with PE. Of the patients sampled, 86% were not diagnosed with PE despite having increased D-dimer values. No specific trends in the relation between elevated D-dimer levels and a positive PE diagnosis could be identified at the significance level of 0.05; a Chi-square test of independence indicated (χ^2^ [1, *N* = 995] = 1.84, *p* = 0.175).

**Conclusion:**

No strong relationship between elevated D-dimer levels in the blood and a positive yield of PE after CTPA; was found hence, clinical decision rules for PE workups need refining, especially to limit unnecessary CTPA referrals in this setting.

**Contribution:**

The findings suggest that PE workup at the private practice should be revised to improve the quality of service.

## Introduction

Pulmonary embolism (PE) is one of the most common heart and blood vessel diseases worldwide. This is a severe and acute disease with potentially fatal consequences. According to Osho and Dudzinski (2022), PE is the third most common cause of cardiovascular death in the United States, after stroke and coronary artery disease. Between 200 000 and 500 000 patients are predicted to be diagnosed with PE annually in the United States (Kaizer-Permanente Washington 2022). In addition, PE results in mortality and haemodynamic instability (AL-Rammah, Alohaly & Albatsh 2016; Moore et al. 2018). Because anticoagulation increases the risk of bleeding and because treatment and monitoring are expensive, PE must be ruled out (Patel et al. 2020). Acute PE can cause dysfunction and changes in pulmonary circulation in the respiratory system; therefore, timely diagnosis and treatment of PE is imperative to manage this potentially life-threatening condition.

Pulmonary embolism has a non-specific clinical appearance, which complicates diagnosis and correctly and promptly identifying the condition in the emergency department (Salehi et al. 2021). A patient is likely to undergo a lower extremity ultrasound to rule out deep vein thrombosis, a computed tomography pulmonary angiography (CTPA) to confirm the existence or absence of PE, and chest X-ray imaging, in addition to the laboratory test, to assess the lung function (Moore et al. 2018). The gold standard for diagnosing PE is computed tomography (CT) imaging, which is widely accessible, offers extremely precise, non-invasive pictures of the anatomy and is highly specific and sensitive for PE (Pathak, Rendon & Muthyala 2011). As a result of the advantages of CT imaging, the demand for CTPA procedures to confirm PE has increased, and both government and private radiology practices frequently perform CTPA examinations on patients who are suspected to have PE. In diagnosing PE, two notable concerns associated with CTPA imaging are (1) radiation exposure risk and (2) adverse responses to using an image-enhancing contrast medium (CM) (Brenner & Hall 2007). The high radiation doses associated with CTPA may have unintentional stochastic and/or deterministic effects. When CM is administered, allergic reactions can occur, including anaphylactic shock, skin reactions (redness and swelling), nausea, metallic taste in the mouth and, in extreme situations, renal failure (Naufal 2012).

The coronavirus disease 2019 (COVID-19) global pandemic caused a noticeable spike in requests for CTPA procedures in Africa. This spike resulted from the clinical presentation of the COVID-19 viral infection, which is linked to severe respiratory systemic inflammation and an increased risk of venous thrombosis and, consequently, PE (Masselli et al. 2021; Rothzinger et al. 2020). Individuals with severe manifestations of COVID-19 are at a higher risk of PE and venous thrombosis (Konstantinides 2020). Given the large number of COVID-19 patients who were seeking medical attention at the time, the International Society on Thrombosis and Haemostasis (ISTH) suggested using laboratory blood tests, such as the D-dimer test, prothrombin time and platelet count, to identify patients who required hospital admission (Spyropoulos et al. 2020).

An essential diagnostic laboratory test for PE diagnosis is the measurement of blood D-dimer levels. Elevated intravascular D-dimer levels indicate thrombus formation (Szigeti 2014; Weitz, Fredenburgh & Eikelboom 2017). During thrombus development, the fibrinolytic system first cleaves fibrinogen by thrombin to form fibrin monomers, which are then used to produce D-dimers (Tuck et al. 2021). The next step involves the formation of polymers by factor XIIIa, which creates crosslinks between adjacent D-domains. When fibrin clots break down, plasmin releases the D-dimer molecules. Initially, laboratory tests could not distinguish between fibrinogen and products of fibrin degradation; however, by using monoclonal antibody-based assays, D-dimers could be measured (Tuck et al. 2021). Later, improvements were made to the D-dimer tests, which included enzyme-linked immunofluorescent immunoassays (EIFAs), microplate enzyme-linked immunosorbent assays (ELISAs) and latex agglutination quantitated tests (Sumney & Whiteman 2007).

In the research setting, the standard protocol for the workup for patients with suspected thrombosis involves performing D-dimer tests routinely; patients with elevated D-dimer levels and chest pain are then sent for CTPA. The researcher noticed that, during the COVID-19 pandemic, there was an increase in the number of patients with positive D-dimer tests and chest pain being sent for CTPA. The number of patients who underwent CTPA and who were retrieved from the Picture Archiving and Communications System (PACS) was substantially more for the 2020 year of COVID-19 (*n* = 813) than the number of patients in 2019 (*n* = 666), 2018 (*n* = 580) and 2017 (*n* = 551) (PACS, accessed 13 February 2021). A review of the PACS data indicates that few of these patients were ultimately diagnosed with PE. This observation – that patients with elevated D-dimer levels who underwent CTPA did not have PE – indicates that referral for CTPA should not be based solely on increased D-dimer levels. This approach is questioned, as CTPA could give rise to excessive radiation exposure, allergic reactions to CM and contrast nephropathy and place a significant financial burden on both the patient and the healthcare system (Sun & Lei 2017). This begs the question, What is the relation between increased D-dimer levels and CTPA confirmation of PE? This study was, therefore, undertaken to establish the relationship between elevated D-dimer levels and positive CTPA for all patients with chest pain and suspected PE.

## Research methods and design

### Study design, population and sampling

A retrospective descriptive study design was adopted to accumulate quantitative, numerical data from the PACS at the research setting to retrieve the required information regarding CTPA imaging performed to confirm PE from January 2019 to December 2020. The data were analysed generally and described as frequencies, central tendency and dispersion. The numerical data were analysed using statistical procedures (Polit, Beck & Hungler 2001). The radiology reports on the selected CTPA examinations were scrutinised to ascertain if a relation existed between a patient’s elevated D-dimer level and a positive diagnosis of PE with CTPA.

Data were collected at a private diagnostic radiology practice in Bloemfontein, Free State province, South Africa. Data were collected retrospectively from the PACS. Clinical information was taken from the CTPA referral letters, including references to chest pain, D-dimer values, previous history of PE and COVID-19 diagnosis. The study population comprised all patients referred for a CTPA examination for suspected PE from January 2019 to December 2020. After applying the inclusion and exclusion criteria, a sample of 1219 patients was included in this study. These patients were male and female patients aged 18–85 years who had been referred for CTPA. Patient data that lacked information relating to the radiation dose and CM amounts were excluded.

### Data capturing

Data collection was executed in three phases. In Phases 1 and 2, a data-capturing instrument (DCI) was designed to capture available patient data from the PACS. In Phase 3, the data were statistically analysed to determine the relation between elevated D-dimer levels and the confirmation of PE using CTPA.

### Data-capturing instrument

An extensive literature review was undertaken to establish the information that the DCI should capture. A two-step approach was followed. Firstly, the data-capturing variables of the instrument (biographical and clinical indicators, CTPA imaging condition values) were identified and used to construct a draft instrument. The draft DCI included the patients’ clinical indicators (Polit et al. 2001), namely, chest pain, increased D-dimer level, previous history of PE, COVID-19 positive, 10-day post-COVID-19 test, COVID-19 outpatients, COVID-19 hospitalised patients and COVID-19 ventilated patients. The imaging condition values of all patients’ total examination dose reports, indicated as the dose length product in mGy/cm, computed tomography dose index (CTDI) volume, scan time, scan range or area (also known as scan length), and the total number of sequences performed at the end of all CTPA examinations, were included in the DCI (Newman et al. 2012). Secondly, the PACS was assessed to determine the different data categories available for the DCI to answer the research question.

The draft DCI was piloted to determine if it contained suitable variables for capturing the data (Polit & Beck 2008). A login code was used to access the CTPA patient data on the PACS. For the pilot study, 10 patients were randomly selected from the PACS. The data of each patient were scrutinised to ensure that a referral letter and a radiology report were present before the draft DCI was used to capture data. If the data were incomplete, another patient was randomly selected; this also applied when patients were selected for the main data collection. During the testing of the DCI, it was found that all relevant variables were present and that the DCI could be used to capture the patient data for this study effectively. The data of the 10 patients of the pilot study were included in the main study (Botma et al. 2010; Polit & Beck 2008).

For the main data collection, the CTPA patient data were systematically assessed, one by one, to ascertain if they complied with the inclusion and exclusion criteria. All patients with compliant data were awarded unique numbers and listed separately to ensure the data could be verified accurately. The DCI sheets were printed separately to capture individual data for compliant patients. No personal information of any patient was recorded, to ensure anonymity and confidentiality of the data.

### Data analysis

After collecting and capturing the data, the data were statistically analysed using SAS Version 9.4 software. Summary statistics were calculated for the variables, namely, means, percentages and ranges. Given that the data are categorical and the observations were independent, Chi-square tests of independence were performed to assess if an association between elevated D-dimer levels in the blood and a positive PE diagnosis after CTPA existed. The Chi-square tests were performed using a level of significance of α = 0.05. In addition, a goodness-of-fit Chi-square test was performed to assess the association between the observations of men and women diagnosed with PE at a significance level of α = 0.05. These tests were performed on different clinical indicator groups composed of D-dimer and PE levels for the sample population and several subpopulations.

### Ethical considerations

Ethics approval was granted by the Health Sciences Research Ethics Committee at the University of the Free State (UFS-HSD 2021/12809). In addition, written consent was obtained from the practice manager of the participating practice. All data were captured retrospectively from the PACS; therefore, no consent was required from patients. To ensure patient privacy and confidentiality, the patients’ data were anonymised once it had been extracted from the PACS system.

## Results and discussion

The clinical information statistics for the sample population (*N* = 1219) gathered from the PACS using the eight clinical indicators were analysed to establish if a relation existed between elevated D-dimer levels and a positive diagnosis of PE with CTPA. After CTPA had been performed, the data revealed that only 16.7% of cases had a positive PE. However, a goodness-of-fit Chi-square test revealed that fewer male patients (*n* = 85) were diagnosed with PE than female patients (*n* = 119) at α = 0.05 (χ^2^ [1, *N* = 204] = 5.67, *p* < 0.017). The clinical information statistics revealed that 3.9% of the patients had chest pain explicitly specified on their referral letters for CTPA (Table 1). Similarly, a previous history of PE was recorded for a small number of patients (5.7%). In contrast, over one-third of the patients (35.8%) had elevated D-dimer levels indicated on their CTPA referral letters. Approximately one-fifth (20.9%) of the patients were COVID-19-positive, of whom most were hospitalised (69.9%). A small number of hospitalised patients required ventilation (1.9%).

**TABLE 1:** Number of patients for the different clinical indicators.

Patient clinical indicator	Number of patients presenting with the clinical indicator	%	Number of patients presenting without the clinical indicator	%	Total number of patients
Chest pain	47	3.9	1172	96.1	1219
Elevated D-dimer level	437	35.8	782	64.2	1219
Previous history of PE	70	5.7	1149	94.3	1219
COVID-19 positive	33	20.9	125	79.9	158
Post 10 days COVID-19 test	81	51.3	77	48.7	158
COVID-19 outpatient	15	9.5	143	90.5	158
COVID-19 hospitalised patient	110	69.6	48	30.4	158
COVID-19 ventilated patient	3	1.9	155	98.1	158

PE, Pulmonary embolism; COVID-19, coronavirus disease 2019.

**FIGURE 1:**
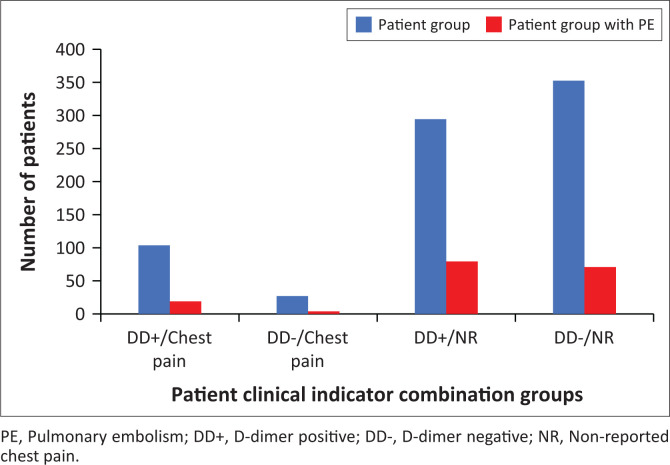
Pulmonary embolism occurrence in different patient clinical indicator combination groups of the sample population.

The relation between elevated D-dimer levels and a positive PE diagnosis was also studied in three population groupings (Figure 1). One of the subpopulations is a population that excluded COVID-19-referred patients (*n* = 955); another subpopulation comprises patients with prehistory of PE (*n* = 106), which excludes patients referred because of COVID-19. The relation between elevated D-dimer levels and a positive diagnosis of PE was also analysed for the subpopulation of COVID-19 referral patients (*n* = 158). No specific trends in the relation between elevated D-dimer levels and a positive PE diagnosis could be identified for the PE-positive patients in the clinical indicator combination groups.

Furthermore, patients who received a positive PE diagnosis after CTPA represented all clinical indicator groups. The percentage of patients diagnosed with PE in the small clinical indicator combination group with chest pain and negative D-dimer tests (*n* = 59) exceeded 50%; in contrast, the percentage of patients diagnosed with PE in the other three groups was less than 20%.

A Chi-square test of independence was performed to establish if different patient groups, classified in terms of combinations of clinical indicators with positive or negative PE diagnosis, were related. The test revealed significant results at α = 0.05 (χ^2^ [3, *N* = 1219] = 58.85, *p* < 0.0001). Table 2 provides a contingency table of the Chi-square test and shows the different clinical indicator groups and PE diagnosis. Four clinical groups, each with a different combination of D-dimer positive or D-dimer negative with either positive or negative PE diagnoses, were subjected to a Chi-square test of independence to determine whether a relationship existed between D-dimer levels and PE diagnoses. According to the results of this test, a significant relationship between the different categories at α = 0.05 (χ^2^ [1, *N* = 995] = 1.84, *p* = 0.175) could not be established.

**TABLE 2:** Contingency table of Chi-square test results for the sample population.

Patient clinical indicator combination group	PE diagnosis		
	PE positive	PE negative	Row totals
Chest pain + D-dimer positive	23 (24.43) [0.08]	123 (121.57) [0.02]	146
Chest pain + D-dimer negative	31 (9.87) [45.20]	28 (49.13) [9.09]	59
Non-specified + D-dimer positive	60 (76.65) [3.62]	398 (381.35) [0.73]	458
Non-specified + D-dimer negative	90 (93.05) [0.10]	466 (462.95) [0.02]	556
Column totals	204	1 015	-

**Total**	-	-	**1219**

PE, Pulmonary embolism.

The data from the sample population of 1219 patients and the subpopulations were analysed, and the results do not indicate a strong relation between elevated D-dimer levels and positive PE diagnoses with CTPA. In many radiology practices, the gold standard for PE diagnosis is through the highly specific and sensitive imaging procedure of CTPA (Doğan et al. 2015; Pathak et al. 2011; Youssf et al. 2014). When patients present with elevated D-dimer levels in the blood, it is standard practice to refer these patients for CTPA to rule out PE (Van Belle et al. 2006). However, PE is not always confirmed in these referred patients, which stimulates the question of whether patients with elevated D-dimer levels should be routinely referred for CTPA and face the risk of high radiation doses and ionic CM administration associated with CTPA imaging (Alshumrani, Al Bshabshe & Mousa 2021). Understanding the relation between elevated D-dimer levels and a positive PE diagnosis with CTPA may influence current referral practices for patients with elevated D-dimer levels.

### Pulmonary embolism

The high sensitivity and specificity of CT as a diagnostic modality have led to the routine use of CTPA to diagnose PE in patients worldwide. However, growing evidence suggests CTPA is overused if PE is suspected in patients (Salehi et al. 2021). This increased use of CTPA, especially in emergency departments, has brought about concerns about radiation exposure and adverse reactions to CM (Sun & Lei 2017).

This retrospective South African study, conducted at a single private radiology practice in Bloemfontein, reveals that less than 20% (16.7%) of more than 1000 patients were positively diagnosed with PE after CTPA. This positive yield of PE falls at the lower end of the suggested acceptable range (15.4% – 34.7%) prescribed by the Royal College of Radiologists (United Kingdom) (Vrettos et al. 2017). A similar result was found in a study conducted at two hospitals in the United Kingdom, where the positive yield of PE was 14% for 236 CTPA scans. Similarly, in North America, the positive yield of PE, specifically in emergency departments, is also low (8%) (De Wit et al. 2022). This study also reveals that more female patients were diagnosed with PE than male patients, in contrast to the study of Turetz et al. (2018), who found PE more prevalent in male patients.

### Relation between elevated D-Dimer levels and pulmonary embolism with computed tomography pulmonary angiogram

The associated and possibly fatal risks of PE and the cost of monitoring necessitate the timeous and effective diagnosis of PE. Today, D-dimer laboratory assays are extensively used in hospitals and private practices to identify a PE risk, because elevated D-dimer levels indicate thrombus formation (De Wit et al. 2022; Weitz et al. 2017). Patients with positive D-dimer tests are usually referred for a CTPA to evaluate for PE (Van Belle et al. 2006). However, elevated D-dimer levels are not only indicative of PE, but also of sepsis, malignancy, pregnancy, myocardial infarction and recent surgery (Righini et al. 2014) and, more recently, COVID-19 (Masselli et al. 2021; Yong et al. 2020). The association between elevated D-dimer levels and several clinical conditions could contribute to the increased use of CTPA to exclude PE. This notion is supported by a study that was conducted by Kubak et al. (2016), who found a positive yield of 26.3% for PE after CTPA examinations of 822 patients with elevated D-dimer levels, using a higher threshold value for elevated D-dimer cutoff, of 0.9 mg/L. A further contributor to the relatively low yield of positive PE after CTPA could be physicians who seldom use decision rules after D-dimer testing because of their complexity and credibility (De Wit et al. 2022). In this study, 83 of the 604 patients (13.7%) with elevated D-dimer levels were diagnosed with PE after CTPA. Similarly, in the COVID-19 patient group, 10.4% of patients with elevated D-dimer levels had positive PE diagnoses. Therefore, the outcome of this study supports the notion that a strong relationship between elevated D-dimer levels and a positive PE yield could not be established.

After analysing the sample population of 1 219 patients and several subpopulations, it was not possible to identify a strong relation between elevated D-dimer levels and a positive PE diagnosis with CTPA. Overall, the number of patients in the sample population of this study with positive diagnoses of PE was relatively small – only 16.7%. Of these patients, approximately 50% had elevated D-dimer levels, which was also true for the subpopulation that excluded COVID-19 referral patients. In contrast, a few patients (< 20%) of the subgroup with elevated D-dimer levels had positive diagnoses of PE. These results show that increased D-dimer levels were present in almost 40% of patients (*n* = 204) who were diagnosed with PE (*n* = 83). However, out of the 604 patients with increased D-dimer values, almost 14% had PE diagnoses (*n* = 83). This indicates that 86% of patients were not diagnosed with PE, despite having increased D-dimer values.

After scrutinising the proportion of PE-positive patients in the clinical indicator combination groups, no specific trends in the relation between elevated D-dimer levels and positive PE diagnoses could be identified. However, a goodness-of-fit Chi-square test revealed that the number of male patients (*n* = 85) diagnosed with PE was significantly lower than the female patients (*n* = 119), at α = 0.05 (χ^2^ [1, *N* = 204] = 5.67, *p* < 0.017). Furthermore, all four clinical indicator groups were presented for all patients who had received positive PE diagnoses after a CTPA. The percentage of patients diagnosed with PE in the small clinical indicator combination group characterised by chest pain and negative D-dimer tests (*n* = 59) exceeded 50%. In contrast, the proportion of patients diagnosed with PE in the other three groups was less than 20%. This outcome substantially impacts the understanding of which diagnostic tests that should be used to decide whether to perform a CTPA and could contribute to the reduction of radiation and prevent the overuse of CT. It is important that other diagnostic methods should be considered, for example, non-contrast pulmonary magnetic resonance angiography (MRA), which has proved to be highly sensitive and specific for identifying proximal pulmonary arteries as the site of PE (Mohammad et al. 2023).

## Limitations of the study

This retrospective research project was limited to a single private radiology practice in Bloemfontein, in the Free State, South Africa. Accessing available patient data on the PACS and applying inclusion and exclusion criteria produced a limited population sample at the radiology practice. Furthermore, data from some patients in the study who had been admitted to the practice during the COVID-19 pandemic were incomplete. Missing data included the absence of patient referral letters, the amount of CM used, radiation dose, and whether a combination of CTPA and CT abdominal examinations was used. Regarding D-dimer levels, threshold values were not recorded, and specific D-dimer levels were absent on some referral letters. If the expertise and experience of the reporting radiologist had been incorporated, interobserver variability between reports could have been possible at the participating private radiology practice in Bloemfontein. This variability hampers the generalisability of the results to other radiology departments.

## Conclusion and recommendations

For a patient cohort of more than a thousand, this study found that approximately 50% were referred for CTPA based on elevated D-dimer levels and related symptoms. The other 50% of patients with normal D-dimer levels were referred for CTPA for symptoms such as chest pain and shortness of breath. Approximately 14% of the D-dimer-positive patient group were diagnosed with PE, whereas in the D-dimer-negative patient group, approximately 20% of the patients were diagnosed with PE. Therefore, no meaningful relation could be determined between elevated D-dimer levels and the diagnosis of PE. These results may imply that a positive diagnosis of PE with CTPA does not always relate to increased D-dimer levels. To minimise unnecessary CTPA referrals in this setting and address potential medicolegal concerns, multi-site larger studies are needed to better understand the role of elevated D-dimer levels as an initial indicator of suspected PE. Additionally, other non-ionising imaging modalities, such as non-contrast pulmonary MRA, should be considered and clinical decision rules for PE workups may need to be adjusted.
